# The Use of a Virtual Online Debating Platform to Facilitate Student Discussion of Potentially Polarising Topics

**DOI:** 10.3390/ani7090068

**Published:** 2017-09-02

**Authors:** Paul D. McGreevy, Vicky Tzioumis, Chris Degeling, Jane Johnson, Robert Brown, Mike Sands, Melissa J. Starling, Clive J. C. Phillips

**Affiliations:** 1 Sydney School of Veterinary Science, The University of Sydney, Camperdown 2006, NSW, Australia; vicky.tzioumis@sydney.edu.au (V.T.); mjstarling@fastmail.com.au (M.J.S.); 2 Sydney Health Ethics, The University of Sydney, Camperdown 2006, NSW, Australia; chris.degeling@sydney.edu.au (C.D.); jane.johnson@sydney.edu.au (J.J.); 3 Biz Hub Australia Pty Ltd., 1 Northcote St, Torrensville 5031, SA, Australia; robert.brown@bizhub.com.au (R.B.); mike.sands@bizhub.com.au (M.S.); 4 Centre for Animal Welfare and Ethics, School of Veterinary Science, University of Queensland, Gatton 4343, QLD, Australia; c.phillips@uq.edu.au

**Keywords:** discussion tool, ethical frameworks, safe learning, online debating, virtual human continuum

## Abstract

**Simple Summary:**

The Human Continuum is a classroom exercise for secondary and tertiary students that offers a cooperative/cognitive learning strategy, requiring students to identify and commit to a position in response to a stimulus question. The purpose of the current submission is to contextualize and introduce online, a virtual human continuum for teaching purposes. It describes an innovative tool design while also reporting its use for a particular cohort: a small group of veterinary science students.

**Abstract:**

The merits of students exchanging views through the so-called human continuum exercise (HCE) are well established. The current article describes the creation of the virtual human continuum (VHC), an online platform that facilitates the same teaching exercise. It also reports feedback on the VHC from veterinary science students (*n* = 38). First-year Doctor of Veterinary Medicine students at the University of Sydney, Australia, trialed the platform and provided feedback. Most students agreed or strongly agreed that the VHC offered: a non-threatening environment for discussing emotive and challenging issues; and an opportunity to see how other people form ideas. It also made them think about how to express their ideas and make arguments; and left them feeling more comfortable about expressing their views using it than they would discussing ideas face-to-face (98%, 84%, 79% and 76%, respectively). All respondents agreed or strongly agreed that the VHC encouraged them to consider other opinions. These data suggest that the transition of the HCE to an online platform facilitates dialogue on difficult ethical issues in a supportive environment.

## 1. Introduction

The Human Continuum (HC) is a classroom exercise for secondary and tertiary students that offers a cooperative/cognitive learning strategy requiring students to identify and commit to a position in response to a stimulus question. This sometimes relates to an ethical dilemma, but may just test student understanding of a complex issue, with peer discussion being a valuable learning tool. Two extreme points of view are identified, each associated with the ends of a line. The line may be imagined or drawn on the floor or ceiling with arrows at each end and a midpoint identified. In response to a stimulus question, students identify and occupy their preferred position on this line and then debate with those holding a similar viewpoint (i.e., their neighbors). Students may be numbered and one half asked to exchange places with those holding opposing views, enabling them to discuss alternative positions and reconsider their views. Students are encouraged to reposition themselves on the line, as they wish, between discussions [1] (Wormeli, 2005). The exercise is a good device to stimulate thinking and learning from peers, but it has limitations. For instance, students may follow group leaders whom they want to impress; or they may be unwilling to reveal their true beliefs to others, particularly if these happen to be controversial, unpopular positions that cast them in an unfavorable light (e.g., male students may be reluctant to admit a sensitivity to animals [2] (pp. 269–272) (Paul & Podberscek, 2000). To address these limitations, the merits of a virtual platform were considered.

The current article focuses on an innovative tool design while also reporting its use for a particular cohort: a small group of veterinary science students. The purpose of the current submission is to contextualize and introduce online, a virtual human continuum for teaching purposes, not to present a detailed analysis of the student feedback. Nevertheless, the feedback from tertiary veterinary science students is presented.

## 2. Background on the Need for an Online Debating Platform

An online, virtual human continuum (VHC: a software package called Chatterbox™) was developed to help students acquire and improve argument and critical assessment skills on polarizing topics and could be used to promote engagement by participating schools. The main objective was to use the tool as an introduction to illustrate the wide range of views and the complexity and difficulty of making decisions on issues, and to help students to express justifications for their positions using ethical frameworks and principles [3,4,5], (pp. 38–50) (Entwistle, 2009; Mullan & Main, 2001; Jagger, 2013). We anticipated that online delivery could facilitate discussion and interaction across schools and avoid face-to-face peer pressure, helping students learn to take an ethical stance and to justify this, to make and refine arguments in response to challenges by their peers from a diversity of backgrounds, and to listen to and assess the values and positions of others [6] (pp. 439–457) (Jonassen and Kim, 2009).

The VHC was developed as an opportunity to show tertiary students how other people (in this case, anonymous peers) form ideas when discussing emotive and challenging issues in a non-threatening environment. Like other online forums, it works best with a moderator, who ensures that all participants have an opportunity to exchange ideas without causing offense to others. The VHC exercises are intended to encourage students to appreciate strong and well-constructed arguments and to inspire them to research the facts that underpin discussions on animal welfare [7] (pp. 43–46) (Bauer & Ogilvie, 1996). Additionally, the exercises are designed to inspire students to understand frameworks that underpin debates on animal ethics and to consider the opinions of others.

A further educational objective that may be achieved with the VHC is the improvement of students’ communication skills [8] (pp. 2828–2834), [9] (pp. 1–17) (Orr, 1996; Adams & Frankel, 2007). The VHC exercises are designed to require students to process ideas under the pressure of a finite time limit and to think and respond quickly. We predicted that this requirement would stimulate a rapid flow of responses and avoid participants becoming defensive. The VHC is predicated on the notion that students feel reasonably comfortable discussing controversial issues with their classmates, but that anonymity and the flexibility to change one’s position increases this comfort. As such, it is anticipated that students may be more comfortable expressing their views using the VHC than they are discussing ideas face-to-face in the classroom. That said, at the outset of any VHC session, students are counselled that although other participants may not be able to identify them, all conversations are logged and monitored by the moderator. Ultimately, the VHC has been designed to encourage students to think about how they express their ideas and make arguments. It is expected that this will prepare students to discuss controversial issues with people they do not know in ways that require ethical reflection, and will promote ethical practise [10] (pp. 79–82) (Kong, 2015).

## 3. Practical Aspects of the VHC

Participants are invited to align themselves to one of two polarising statements in response to a stimulus question (generally supported by a link to background reading material). Depending on their view, and the aim of the instructor or exercise, participants are assigned to conversations with other participants, after which they are invited to revisit their position. The precise length and format of the Chatterbox™ session can be determined by the moderator. Participation in the VHC forces students to take a position along a continuum but allows them to alter their position in the light of what they have heard from their peers. It offers teachers the flexibility to pair students with their nearest neighbor on the continuum (e.g., as a warm-up exercise) so that they are not initially confronted with a peer who holds diametrically opposite views. Alternatively, the teacher (or moderator) can pair participants with those farthest from them on the continuum, or simply pair them randomly.

**Figure 1:**
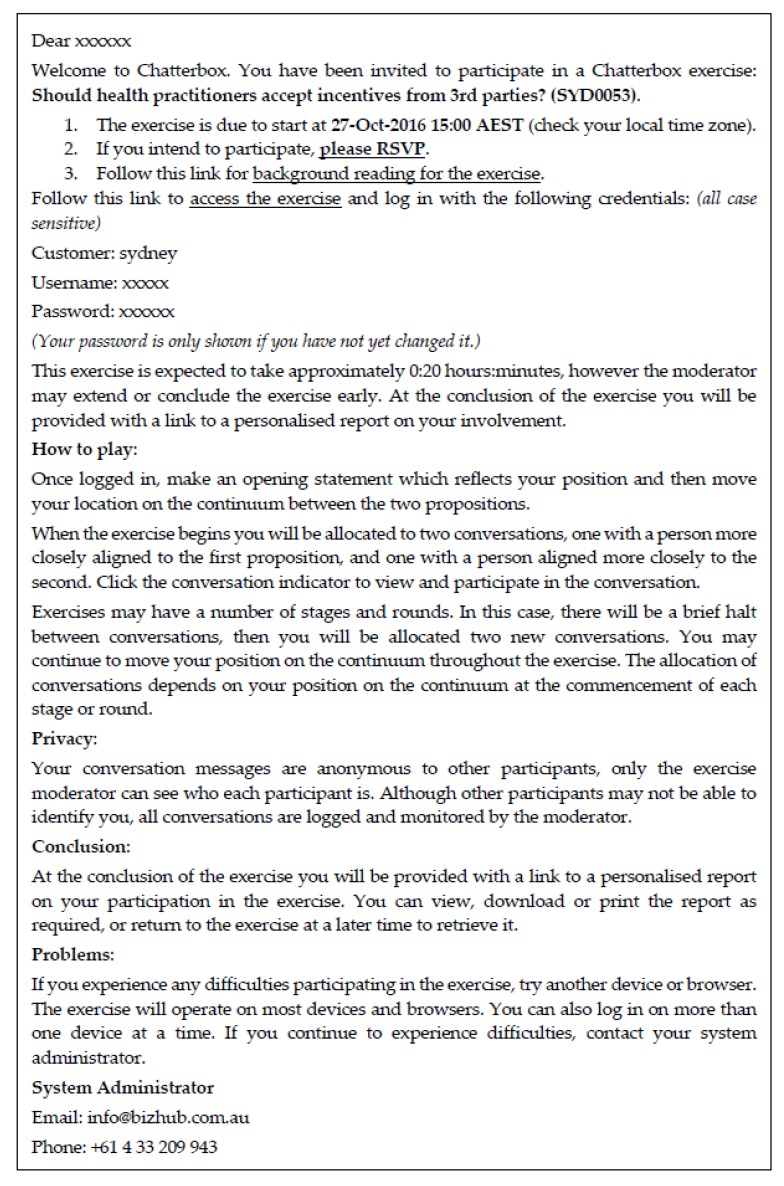
An example of the introductory email for a virtual human continuum (VHC) session.

**Figure 2:**
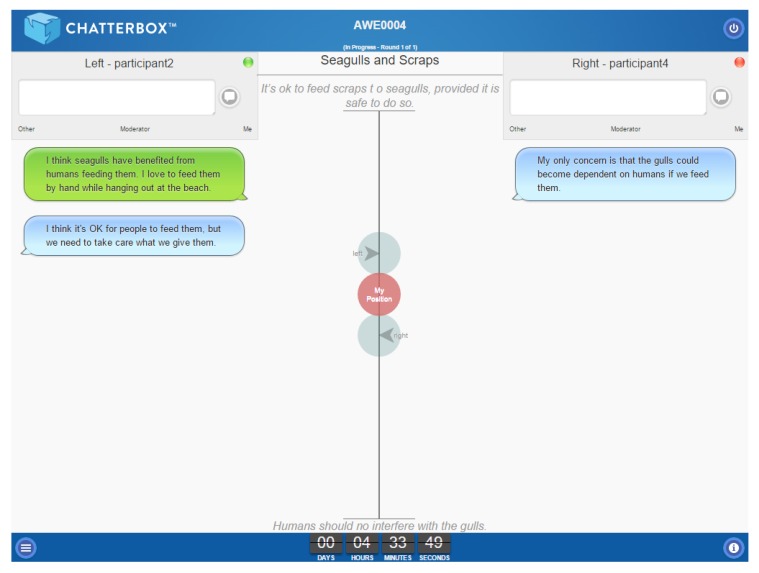
A screen grab from an introductory video to Chatterbox™, available on the One Welfare portal: http://onewelfare.cve.edu.au/chatterbox.

These steps involved in organising the typical Chatterbox™ session are as follows:

1. The moderator sets up the VHC exercise in the Chatterbox™ system. Before the exercise commences, the moderator chooses the length of the exercise (i.e., the number and duration of rounds), and how the conversations are allocated (nearest neighbor, a peer at the opposite end of the spectrum; or randomly).
2. The moderator sends email invitations (through the Chatterbox™ software) to the relevant participant cohort. The email contains details of the question or issue to be discussed, the time that the exercise is scheduled to begin and how long it is expected to last. It also contains links to accept the invitation, to background reading, to access the exercise and login details, along with brief instructions on how to engage in the exercise (see Figure 1).
3. Once participants have entered the VHC exercise they are invited to align themselves to one of two polarising statements (that is, position themselves on the continuum) and each participant is allocated to conversations with two peers (one to their left and one to their right along the continuum). While participants wait for the exercise to begin they are given the opportunity to write an opening statement that summarises their position or viewpoint on the issue.
4. Once the exercise is underway, each participant holds a conversation with two peers for the duration of the round. The moderator has the option to broadcast messages to the entire cohort or to particular conversations while the exercise is underway. A countdown clock is located at the bottom centre of the screen and a warning alerts participants to the final minute of a round. The moderator can extend or conclude the exercise early.
5. Once a round is over, participants see a message on screen indicating that they should await the next round to begin. At this point, the moderator has the option to change how participants are allocated to conversations, and participants have an opportunity to reposition themselves on the continuum.
6. When the exercise ends participants are emailed a summary of all of their conversations (see Figure 2):

An example of a screen grab from a round within a VHC session appears in Figure 2. An introductory video to Chatterbox™ is available on the One Welfare portal: http://onewelfare.cve.edu.au/chatterbox.

**Figure 3:**
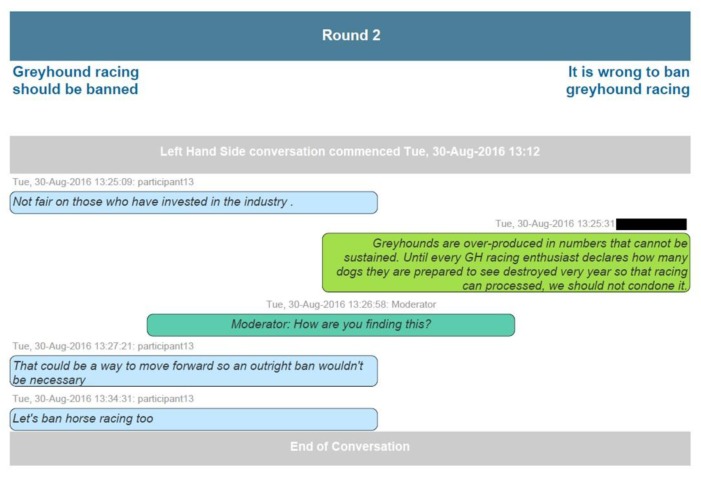
A screen grab from a summary of participation in a VHC session.

An example of a screen grab from a summary of a VHC session appears in Figure 3. 

## 4. Technical Specifications of the VHC Software

We used a cloud-based system (hosted by a provider, rather than in a physical location owned by the user) that realizes the “human continuum” learning strategy. This software supports multiple concurrent instant-message conversations, using criteria defined by an instructor for that exercise, on predefined topics, coordinated by a moderator. Participants respond to an email invitation to “attend” on a “Bring Your Own Device” (BYOD) basis, and nominate a location on a graphical continuum by sliding an indicator within their browser. The system uses this location to allocate participants to conversations, with each participant allocated two conversations. Crucially, the system maintains anonymity for all participants, however the moderator is able to identify participants, review and engage in all conversations, track the use of specified keywords and send broadcast directives to the entire group. During an exercise, students may relocate their position on the continuum, reflecting a change in their views after their conversations. The software supports exercises with multiple topic stages and multiple rounds within each stage. At the close of the exercise, students and moderators retrieve a full transcript of their conversations and graphical depictions of their relative movements along the continuum.

Exercises can be run with cohorts of participants from within or across lecture groups, universities and non-student groups or any member of the public who is issued a system credential. Exercises can be run over any period, from minutes to months, with small or large cohorts. The system allows for the creation and re-use of exercise templates and pre-defined participant cohorts, with data segmentation available for discrete “Schools” as required.

The software is implemented as a Java EE application using the open source Skyve Enterprise Platform created by Biz Hub Australia (www.bizhub.com.au). The Skyve Enterprise Platform implements the Skyve software development methodology (www.skyve.org). All Skyve applications are operating system, browser and database independent and support concurrent use with almost any internet-capable device.

While the Skyve Enterprise Platform is open source, the VHC software (built using the Skyve Platform) is not itself open source—as the intellectual property was created for, paid for and therefore owned by One Welfare. One Welfare may decide to “open” the source at some later date or grant licences as it sees fit.

Currently, the VHC software is configured for the nominated Australian and NZ vet schools—where each school has their own “tenancy” or data segment—providing the ability to own their own data while using the shared software. The software can be made available to additional “tenants” via configuration. Additional class groups, supervisors and participants can be granted access within a tenancy segment by the administrator of each tenancy (and other users to whom this privilege has been granted by the administrator).

## 5. Student Evaluation of the VHC

Students (*n* = 53) from the first-year cohort of a four-year Doctor of Veterinary Medicine degree at the University of Sydney were given feedback evaluation forms. These forms were approved by the University of Sydney Human Research Ethics Committee (Approval number 2014/739), and distributed in hard copy form to students using the VHC. This questionnaire used a five-point Likert scale of agreement to gauge the extent to which the VHC met the original objectives of the project.

## 6. Results

**Figure 4:**
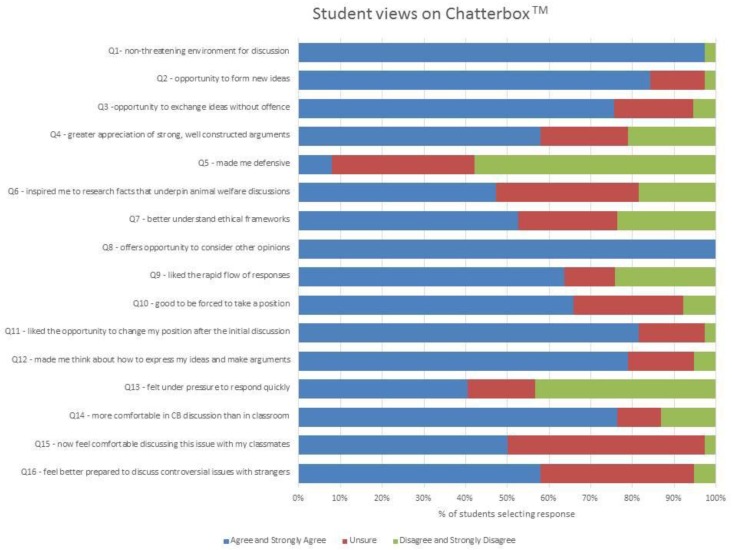
Student feedback on Chatterbox^TM^.

Of this cohort, 38 students (72%) responded. Responses were collated and are presented in a condensed format (with “agree” combined with “strongly agree” and “disagree” combined with “strongly disagree”) in Figure 4.

All respondents (100%) agreed or strongly agreed that Chatterbox™ offered the opportunity to consider other opinions; 98% of respondents agreed or strongly agreed that the VHC activity offered a non-threatening environment for discussing emotive and challenging issues; 84% agreed or strongly agreed that it offered an opportunity to see how other people form ideas; and 79% agreed or strongly agreed that it made them think about how to express their ideas and make arguments. Some 76% agreed or strongly agreed that they felt more comfortable expressing their views using VHC than they would discussing ideas in the classroom. Conversely, 21% of students disagreed that the VHC exercise encouraged them to appreciate strong and well-constructed arguments; and 24% disagreed that the VHC exercise inspired them to understand frameworks that underpin debates on animal ethics.

## 7. Example of Assignments Linked to the VHC Exercises

A variety of assessment-task templates can be linked to a VHC exercise. These templates have been adapted from those used for face-to-face Human Continuum activities. We propose that templates deployed should represent a range of assessment tasks for any cohort of students. For veterinary students, the assessment tasks can address learning outcomes that lead to the development of Day One Competencies relating to Animal Welfare and Ethics (AWE). For the purpose of teaching Australian and New Zealand veterinary students, we favour assessments that can be linked to Royal College for Veterinary Surgeons (RCVS) Day One Competencies. However, we recognize that each university will have its own version of learning outcomes for AWE.

We developed a reporting exercise for students who have recently finished the VHC exercise and have received the summary of their discussions. Students were asked to submit a report of their discussions with their peers. The framework for these reports was as follows:

1. A succinct description of the dilemma.
2. A list of the issues, identifying the relative importance of each.
3. The relevant values, ethical and moral positions.
4. The ethically defensible conclusion(s) of the students’ group OR the key obstacles to reaching an ethical conclusion.
5. The student’s reflections on the exercise and areas for further exploration.

## 8. Assignment Criteria

The written submission can be assessed against different criteria, as proposed in Table 1.

Further suggestions for assessment activities are designed to help teachers ensure student engagement by linking the VHC discussion to assessment.

1. Based on your VHC discussion, write a short report that includes (i) a brief description of the dilemma in your own words; (ii) identification of the main ethical issues raised by the dilemma; and (iii) your response to these ethical issues, including a justification of your conclusions.
2. Identify the most compelling/persuasive argument made by someone else in the VHC discussion you engaged in. In your own words, describe the thread of posts in which that argument appeared. Why was the argument compelling/persuasive? Give your best response to this argument.
3. Revisit your posts on VHC and choose what you consider to be the weakest and strongest points you made. Rewrite and improve these posts, making explicit the ethical theory or theories that underpin your position.

## 9. Discussion

We developed a VHC through Chatterbox™ to promote engagement by all the veterinary schools and veterinary faculties in Australia and New Zealand that were participating in a teaching development project. Along with other teaching and learning innovations that have emerged from this collaboration, the VHC represents one option for an online version of the “Human Continuum” learning activity that can be found in the toolbox on the open access *One Welfare* Portal (http://onewelfare.cve.edu.au). It facilitates discussion of difficult and controversial AWE issues in a non-confrontational environment. It also provides an opportunity for including participants from other classes at geographically distant locations, enabling students to discuss with other students who may have different cultural or learning backgrounds from that of their classmates. We can see applications for this innovation beyond Australia and New Zealand and beyond the teaching of animal welfare and ethics. We anticipate that it will be made available through the University of Sydney.

The results of our pilot study confirm that this online version of the “Human Continuum” activity is attractive and conducive to good thinking. As the creators of the tool, we were particularly pleased to note that 76% of the students agreed or strongly agreed that they felt more comfortable expressing their views using Chatterbox™ than they would discussing ideas in the classroom. We acknowledge the need for extra study in the wake of a Chatterbox™ exercise and have offered suggestions for assignments that may help students to develop an ethical stance or justify their views using ethical frameworks or principles.

There is a potential limitation to the approach if contributors to the discussion come from the same educational and ethical background (i.e., veterinary students). All may adopt a similar viewpoint that reflects either their background or what they perceive to be the appropriate position of the veterinary profession. However, the tool does allow the simultaneous participation of students from the eight veterinary schools in a session. These schools may have a different teaching focus (e.g., some emphasize production animals while others have a clear strength in companion animals). Chatterbox™ may reveal differences among schools, both in student backgrounds and teaching philosophy, that could be researched. The discussions could be opened up to a broader group of participants, but we accept that it would be challenging for students if they were confronted (albeit anonymously) with individuals, on either side, who were prepared to defend strongly held extremist views, whether by conviction or through role playing. This may be appropriate in the later stages of their course, to prepare them for their work as veterinarians, when such views may be regularly encountered. Effective moderation is important, so that the organizer intervenes in the case of use of bad language or other inappropriate online behavior. The moderator can monitor the output from participants in real time, looking for keywords (and, for that matter, derogatory or inflammatory terms).

The tool can be used as an introduction to illustrate the wide range of views on issues and the complexity and difficulty of making decisions on issues, before introducing ethical frameworks and ethical decision-making tools. It can help students to express justifications for their positions using ethical frameworks and principles. It is important to consider the extent of information given to students prior to their discussion, which could range from asking them to read a journal article to simply posing a controversial issue to them.

We emphasize that the intention is not to arrive at a general consensus during the exercise but to give students the opportunity to discuss difficult or controversial ethical issues in a non-threatening way. Follow-up discussions can take place in the classroom. Chatterbox™ can be used to illustrate how the same student may adopt different perspectives or ethical frameworks on different issues concerning the use or care of animals. Students will be encouraged to conduct their own research for further information, thus developing the important skills students require to locate information on their own, while refining interpersonal communication skills [11] (pp. 19–21) (Herron, Wolf & DiBrito, 1990). They will also be encouraged to reflect on and change their ethical positions if they hear an argument that helps them rethink their position [6] (pp. 439–457) (Jonassen & Kim, 2009). We anticipate that this tool will be of interest to a wide range of educators beyond AWE and may be of merit for practitioners to reflect upon and discuss ethical dilemmas with anonymised peers [12] (Schon, 1983).

## 10. Conclusions

The VHC reported here is an online version of an HC exercise. Moving an HC exercise to an anonymous online platform was intended to facilitate discussion of potentially controversial topics so that students could express themselves without fear of being judged negatively by their teachers or peers. The student evaluations of the VHC exercise support this, with most students agreeing that the online platform provided a non-threatening environment in which to communicate and listen to opinions on AWE topics. The VHC has the additional advantage of being able to include participants from multiple institutions, increasing the range of opinions on the issues to be discussed. We believe the VHC has potential as a valuable learning tool in communication and discussion in most academic disciplines.

## Figures and Tables

**Table 1:** Suggested framework for student assessment of the VHC exercise.

Attribute	% Marks
The dilemma is accurately articulated.	10
All issues are identified.	10
The priority given to each issue is clearly argued.	10
The values, ethical and moral positions are articulated in a professional and non-emotional manner.	30
The conclusion of the discussion is clearly presented.	10
Any obstacles to reaching a consensus position are clearly presented.	10
Your reflections include any changes in your positon as a result of the discussion.	10
Areas of animal welfare and ethics you wish to explore further are defined.	10
